# Growth-Suppressive and Apoptosis-Inducing Effects of Tetrandrine in SW872 Human Malignant Liposarcoma Cells via Activation of Caspase-9, Down-Regulation of XIAP and STAT-3, and ER Stress

**DOI:** 10.3390/biom12060843

**Published:** 2022-06-17

**Authors:** Mohammed Samsuzzaman, Byeong-Churl Jang

**Affiliations:** Department of Molecular Medicine, College of Medicine, Keimyung University, 1095 Dalgubeoldaero, Dalseo-gu, Daegu 42601, Korea; samsuzzaman238@gmail.com

**Keywords:** tetrandrine, SW872, apoptosis, caspase-9, STAT-3, ER stress

## Abstract

Liposarcoma is a rare and heterogeneous soft tissue malignant tumor and has a significant impact on mortality with a poor prognosis. To date, there is no effective treatment for liposarcoma, whereas surgical resection is only the gold treatment with numerous adverse effects. Here we investigated whether tetrandrine inhibits the growth of SW872 human malignant liposarcoma cells. Of note, tetrandrine at 10 μM vastly inhibited growth and induced apoptosis, as evidenced by increased nuclear DNA fragmentation and sub-G1 population of SW872 cells. Mechanistically, treatment with tetrandrine led to activation of caspase-9/3 in SW872 cells, and z-VAD-fmk, a pan-caspase inhibitor, attenuated the tetrandrine-induced apoptosis and growth suppression in SW872 cells. In addition, tetrandrine treatment resulted in down-regulation of XIAP andSTAT-3 in SW872 cells, and importantly knockdown of STAT-3 caused a significant reduction of the cell survival. Tetrandrine also had abilities to up-regulate not only the expression of GRP78 and ATF-4 but also the phosphorylation of eIF-2α in SW872 cells. In summary, these results demonstrated that tetrandrine has strong growth-suppressive and apoptosis-inducing effects on SW872 cells, which are mediated through control of the intrinsic caspase pathway, down-regulation of XIAP and STAT-3, and triggering ER stress.

## 1. Introduction

Liposarcoma is the second-highest soft tissue aggressive malignant tumor, with at least 30 cases per million annually in the United States [1,2,3,4]. Notably, there are still limitations in diagnosing patients with soft tissue liposarcoma due to malignancy of cell types, tumor size, and anatomical location [5]. Currently, the options for treating of liposarcoma patients are chemotherapy, radiotherapy, and surgery [6]. Although there has a considerable improvement in cancer therapy, a favorable response to conventional treatment in liposarcoma remains unsatisfactory. Therefore, there is still a need for novel, non-toxic, and more effective therapeutic agents to comply with the necessity of liposarcoma patients.

Apoptosis is an essential programmed cell death event to eliminate needless cells from our body, as a part of the homeostasis process during the development of multi-cellular organisms and tumor cells from patients by anti-cancer drugs [7]. Cells undergoing apoptosis are well defined by distinct cellular morphological changes, such as cell shrinkage, plasma membrane blebbing, mitochondrial dysfunction, chromatin condensation, and nuclear DNA fragmentation [8]. Accordingly, many cellular proteins and factors are involved in regulating apoptosis. Among them, caspases play an essential role in the process of apoptosis in response to apoptotic stimuli [9]. Activation of caspases is also known to be regulated by a set of anti-apoptotic proteins, including inhibitors of apoptosis proteins (IAPs) and B cell lymphoma-2 (Bcl-2) family proteins [10,11]. Several studies have further demonstrated that endoplasmic reticulum (ER) stress plays a crucial role in the apoptosis induction of cancer cells [12,13]. A growing body of evidence also illustrates the involvement of numerous signaling proteins and transcription factors, including Janus-activated protein kinases (JAKs) and signal transducer and activator of transcriptions (STATs), in the regulation of cell proliferation, survival, and apoptosis [14,15,16]. 

Tetrandrine, a natural compound isolated from *Stephania tetrandrae*, has been used as a clinical drug in China to treat patients with inflammatory disease, hypertension, autoimmune disorder, silicosis, and cardiovascular disease [17,18]. Many in vitro and in vivo studies also strongly indicate the capability of tetrandrine to induce anti-cancer effects by slowing tumor growth and accelerating survival time [19,20] and by inducing apoptosis in different types of human cancer cells, including HepG2 (liver), A549 (lung), and HCT-116 (colon) [21,22,23]. It also has been shown that tetrandrine inhibits the migration and invasion of SiHa human cervical cancer cells via matrix metalloproteinase-2 (MMP-2) and MMP-9 down-regulation [24], and this natural substance suppresses the metastatic phenotype of prostate cancer cells by negatively regulating Akt/mTOR/MMP-9 signaling pathway [25]. To date, however, tetrandrine regulation of liposarcoma cells is not fully understood. In the present study, we investigated the effect of tetrandrine on the growth of SW872 cells, a human malignant liposarcoma cell line. Here we demonstrate, for the first time, that tetrandrine at 10 μM has strong growth-suppressive and apoptosis-inducing effects on SW872 cells, mediated through control of the intrinsic caspase pathway, XIAP, STAT-3, and ER stress. 

## 2. Materials and Methods

### 2.1. Chemicals and Antibodies

Tetrandrine was purchased from Selleckchem (Houston, TX, USA). DR-5 antibody was bought from IMGENEX (Cambridge, UK). Antibodies for procaspase-9 and procaspase-3 were obtained from Enzo (New York, NY, USA). PARP and eIF-2α antibodies were bought from Cell Signaling Technology (Danvers, MA, USA). Antibodies for XIAP and HIAP were bought from R&D (Minneapolis, MN, USA). Antibodies for Mcl-1, Bak, Bax, STAT-3, phospho(p)-STAT-3, GRP78, and ATF-4, secondary anti-rabbit, anti-mouse, and anti-goat IgG antibodies, and control siRNA and STAT-3 siRNA were purchased from Santa Cruz Biotechnology (Delaware, CA, USA). p-eIF-2α antibody was bought from Abcam (Cambridge, MA, USA). z-VAD-fmk was bought from Calbiochem (Madison, WI, USA). Enhanced chemiluminescence (ECL) Western detection kits were obtained from Advansta (San Jose, CA, USA).

### 2.2. Cell Culture

Human malignant liposarcoma cells [SW872 (CRL-HTB92™) and 93T449 (CRL-3043™)] were purchased from ATCC (Manassas, VA, USA) and maintained at 37 °C in a humidified atmosphere (95% air and 5% CO2) in DMEM and RPMI 1640 media supplemented with 10% heat inactivated-fetal bovine serum (HI-FBS)and 1% penicillin/streptomycin. HaCaT cells, a normal human skin keratinocyte cell line, were kindly provided by professor Young-Wook Ryoo (Department of Dermatology, College of Medicine, Keimyung University) and cultured in DMEM/F12 media supplemented with 10% HI-FBS and 1% penicillin/streptomycin.

### 2.3. Cell Counting and Morphological Analysis

Cells were seeded in a 24-well cell culture plate at a density of 1.5 × 10^5^ cells/mL per well in the final volume of 500 µL. After overnight incubation, cells were treated with or without tetrandrine in the absence or presence of z-VAD-fmk for the designated times and concentrations. At each time point, the number of surviving cells that could not be stained with trypan blue dye was counted using standard phase-contrast microscopy. Morphological changes of the conditioned cells treated with tetrandrine or transfected with siRNA of control or STAT-3 were captured by phase-contrast microscopy equipped with Nixon digital camera. 

### 2.4. Measurement of DNA Fragmentation

DNA fragmentation assay was carried out according to the previously described method [26]. SW872 cells were cultured at a density of 1.5 × 10^5^ cells/mL for 24 h. Cells were treated with or without tetrandrine at the indicated doses in the absence or presence of z-VAD-fmk for 24 h. The conditioned cells were then harvested, washed, and lysed in DNA isolation buffer [50 mM Tris (pH 8.0), 0.5% sarkosyl, 0.5 mg/mL proteinase K and 1 mM EDTA] at 55 °C for 3 h, followed by the addition of RNase A (0.5 μg/mL) and incubation at 55 °C for 18 h. The cell lysates were further centrifuged at 13,000 rpm for 20 min. Genomic DNA was extracted with an equal volume of neutral phenol-chloroform-isoamyl alcohol mixture (25:24:1) and analyzed by electrophoresis on a 1.8% agarose gel. The DNA was visualized and photographed under UV illumination after staining with ethidium bromide (0.1 µg/mL) by a Gel documentation system (Gel Doc-XR, Bio-Rad, Hercules, CA, USA).

### 2.5. Measurement of the Sub-G1 Population by Flow Cytometry Analysis

SW872 cells were seeded overnight and treated with vehicle control (DMSO) or tetrandrine at the indicated doses for 24 h. The conditioned cells were collected, washed with PBS, fixed in ice-cold 70% ethanol, and stored at 4 °C. Cells were then washed with PBS and stained with 1% cold propidium iodide solution containing RNase A (100 μg/mL), propidium iodide (50 μg/mL), 0.1% (*w*/*v*) sodium citrate, 0.1% (*v*/*v*) Nonidet P-40 (NP-40) and incubated for additional 30 min in the darkness. The sub-G1 population of cells was measured with a flow cytometer (FACS Caliber, Becton Dickinson, MD, USA) and CellQuest software. Approximately 10,000 cells were counted for the analysis. 

### 2.6. Preparation of Whole-Cell Lysates

SW872 cells were seeded in a 6-well cell culture plate at a density of 1.5 × 10^5^ cells/mL overnight. Cells were then treated with vehicle control (0.1% DMSO) or tetrandrine at the designated concentrations in the absence or presence of z-VAD-fmk (20 μM)) for the indicated time points. At each time point, the conditioned cells were washed with PBS, and extracted in a modified RIPA buffer (50 mM Tris-Cl (pH 7.4), 0.1% sodium dodecyl sulphate, 150 mM NaCl, 1% Triton X-100, 1 mM EDTA, 1% NP-40, full-name (PIC) (1X), 1 mM EGTA). The cell lysates were centrifuged at 13,000 rpm at 4 °C. The resultant supernatant was retained in a 1.5 mL tube, and its protein concentration was quantified by bicinchoninic acid assay (BCA) protein assay (Pierce, Rockford, Tempe, AZ, USA).

### 2.7. Western Blot Analysis

Proteins (30 μg) were separated by sodium dodecyl sulfate-polyacrylamide gel electrophoresis (SDS-PAGE) and transferred into a nitrocellulose membrane. The membrane was washed with TBS (10 mM Tris, 150 mM NaCl) supplemented with 0.05% (*v*/*v*) Tween 20 (TBST) and blocked with TBST containing 5% (*w*/*v*) non-fat dried milk for 3 h. The membrane was then washed with TBST and incubated with primary antibodies for 20 h at 4 °C, followed by incubation with secondary antibodies coupled to horseradish peroxidase for 2 h. The membrane was washed with TBST, and immunoreactivity of specific proteins was detected by ECL reagents. The expression levels of β-actin were used to assess equal protein loading and relative expression of the target protein of interest.

### 2.8. Reverse Transcription-Polymerase Chain Reaction 

After treatment, total RNA from the conditioned cells was isolated with the RNAzol-B (TelTest, Friendswood, TX, USA). One microgram of total RNA was reverse transcribed using a random hexadeoxynucleotide primer and reverse transcriptase. Single-stranded cDNA was amplified by PCR with the following primers. Primer sequences used for amplification were as follows: XIAP sense 5′-CGTCGATTTTTGTGATGCTCGTCAG-3′; XIAP antisense 5′-GAAGCATTTATCAGGGTTATTGTCTCA-3′; Actin sense 5′-TCATGAAGTGTGACGTTGACATCCGT-3′; Actin anti-sense 5′-CCTAGAAGCATTTGCGGTGCACGATG-3′. The PCR conditions applied were: XIAP, 30 cycles of denaturation at 94 °C for 4 min, annealing at 54 °C for 40 s, and extension at 72 °C for 30 s and Actin, 25 cycles of denaturation at 95 °C for 30 s, annealing at 57 °C for 30 s, and extension at 72 °C for 1 min. Expression levels of actin mRNA were used as an internal control to evaluate the relative mRNA expression of apoptosis-related genes.

### 2.9. Transfection of Small Interfering RNA (siRNA) 

SW872 cells seeded into a 6-well plate at the density of 1 × 10^5^ cells/mL/well were transfected with 100 picomoles (pM) of control or STAT-3 siRNA using Lipofectamine^®^ RNAiMAX Transfection Reagent (Invitrogen, Waltham, MA, USA) for 6 h. After removing culture media, the transfected cells were grown in DMEM containing 10% HI-FBS, followed by incubation for 32 h. The number of surviving cells, which cannot be stained with trypan blue dye, was then counted under the microscope. Data are means ± SE of three independent experiments. 

### 2.10. Statistical Analysis

Cell count assay was carried out in triplicate. Data are expressed as means ± standard errors (SE) from three independent experiments. Statistical significance was determined by One-Way ANOVA (Laerd Statistics, Chicago, IL, USA). All significance testing was based upon a *p*-value of < 0.05.

## 3. Results

### 3.1. Tetrandrine at 10 μM Vastly Suppresses Growth and Induces Apoptosis of SW872 Cells

**Figure 1:**
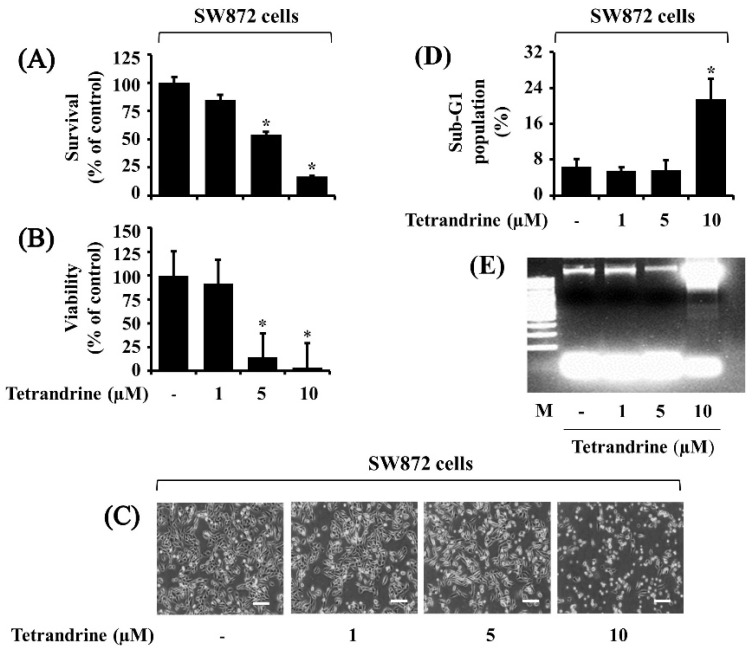
Effects of tetrandrine on the survival, viability, and apoptosis of SW872 cells. (**A**–**E**) SW872 cells were treated with vehicle control (0.1% DMSO) or tetrandrine at different concentrations (1, 5, or 10 μM) for 24 h. The number of surviving cells was analyzed by cell count assay (**A**). The cell count assay was carried out in triplicate. Data are expressed as mean ± SE of three individual experiments. * *p* < 0.05 was compared to the value of tetrandrine-free control at the indicated concentrations. The viability of cells was analyzed by the MTS assay (**B**). The MTS assay was carried out in triplicate. Data are expressed as mean ± SE of three individual experiments. * *p* < 0.05 was compared to the value of tetrandrine-free control at the indicated concentrations. The morphological image of the conditioned cells was captured by a phase-contrast microscope (100×, scale bar = 12.5 μm). Each image is the representative of three independent experiments (**C**). SW872 cells were treated with vehicle control (0.1% DMSO) or tetrandrine at different concentrations (1, 5 or 10 μM) for 24 h, and the conditioned cells were stained with propidium iodide (PI). The apoptotic cells with the sub-G1 phase were detected by flow cytometry analysis (**D**). SW872 cells were treated with vehicle control (0.1% DMSO) or tetrandrine at different concentrations (1, 5, or 10 μM) for 24 h. Genomic DNA from the conditioned cells was extracted and analyzed by 1% agarose gel electrophoresis. M, a DNA size marker (**E**).

Initially, we investigated the treatment effect of tetrandrine at different concentrations (1, 5, and 10 µM) for 24 h on the growth of SW872 cells by using cell count analysis. As shown in Figure 1A, compared with control, treatment with tetrandrine at 5 and 10 μM significantly reduced the survival of SW872 cells. We also determined the treatment effect of tetrandrine on the viability of SW872 cells by using the MTS assay. As shown in Figure 1B, tetrandrine treatment at 5 and 10 μM for 24 h significantly inhibited the viability of SW872 cells. Microscopic observation further confirmed the ability of tetrandrine to inhibit the growth of SW872 cells (Figure 1C). Cells undergoing apoptosis are characterized by an increase in the sub-G1 phase and nuclear DNA fragmentation [7]. As shown in Figure 1D, results of FACS analysis showed that while treatment with tetrandrine at 1 or 5 μM did not increase the sub-G1 population of SW872 cells, that with tetrandrine at 10 μM resulted in a marked accumulation of the cells with sub-G1 phase in SW872 cells. Furthermore, as shown in Figure 1E, data of DNA fragmentation experiments demonstrated that while treatment with tetrandrine at 1 or 5 μM did not induce nuclear DNA ladder in SW872 cells, that with tetrandrine at 10 μM led to induction of genomic DNA fragmentation in the cells. We next determined the effects of tetrandrine on the growth of 93T449 cells, another human liposarcoma cell line, by using cell count and MTS assays. Similar to SW872 cells, treatment with tetrandrine at 5 and 10 μM for 24 h significantly inhibited the survival and viability of 93T449 cells (Supplementary Figure S1A–C). We also examined tetrandrine’s effect on the growth of HaCaT cells, a normal human skin keratinocyte cell line. Results of cell count and MTS assays demonstrated that tetrandrine treatment at doses tested had no or little inhibitory effects on the survival and viability of HaCaT cells, respectively (Supplementary Figure S1D,E).

### 3.2. Tetrandrine at 10 μM Induces Activation of Caspase-9, Caspase-3, and Accumulation of Cleaved PARP in SW872 Cells

**Figure 2:**
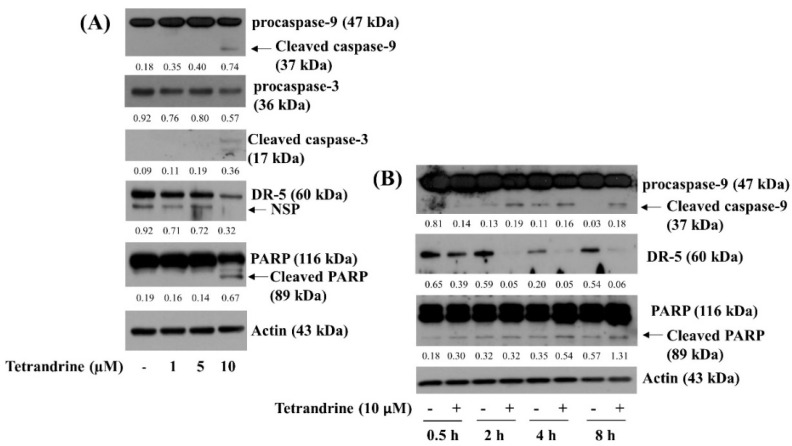
Effects of tetrandrine on the expression of caspases, DR-5, and PARP in SW872 cells. (**A**) SW872 cells were treated with vehicle control (0.1% DMSO) or tetrandrine at different concentrations (1, 5, or 10 μM) for 24 h. Whole-cell lysates from the conditioned cells were prepared and analyzed by Western blotting to measure the expression of procaspase-9, procaspase-3, DR-5, PARP, or β-actin. (**B**) SW872 cells were treated with vehicle control (0.1% DMSO) or tetrandrine at 10 μM for the designated time point. Whole-cell lysates from the conditioned cells were prepared and analyzed by Western blotting to measure the expression of procaspase-9, procaspase-3, DR-5, PARP, or β-actin.

We next examined the treatment effect of tetrandrine at different concentrations (1, 5, and 10 µM) for 24 h on the expression of apoptosis-related markers, such as caspases, DRs, and PARP, in SW872 cells by using Western blotting analysis. In this study, tetrandrine’s ability to induce caspase activation in SW872 cells was assessed by relatively measuring expression (accumulation) levels of inactive pro-form and active (cleaved) form of these enzymes. As shown in Figure 2A, treatment with tetrandrine at 1 or 5 μM did not modulate expression levels of procaspase-9 and procaspase-3 in SW872 cells. However, tetrandrine treatment at 10 μM decreased the expression levels of procaspase-9 and procaspase-3 while increasing cleaved caspase-9 and caspase-3 in SW872 cells. Tetrandrine treatment at the doses tested did not elevate expression levels of DR-5 in SW872 cells. As further shown in Figure 2B, results of kinetic studies demonstrated tetrandrine’s ability at 10 μM to increase expression levels of cleaved caspase-9 and PARP while down-regulating DR-5 expression in SW872 cells over time. Expression levels of control actin protein remained constant under these experimental conditions. Due to solid anti-survival and pro-apoptotic effects on SW872 cells, this 10 µM concentration of tetrandrine was chosen for further studies.

### 3.3. Activation of Caspases Is Crucial for Tetrandrine (10 μM)-Induced Pro-Apoptotic and Anti-Survival Effects on SW872 Cells

**Figure 3:**
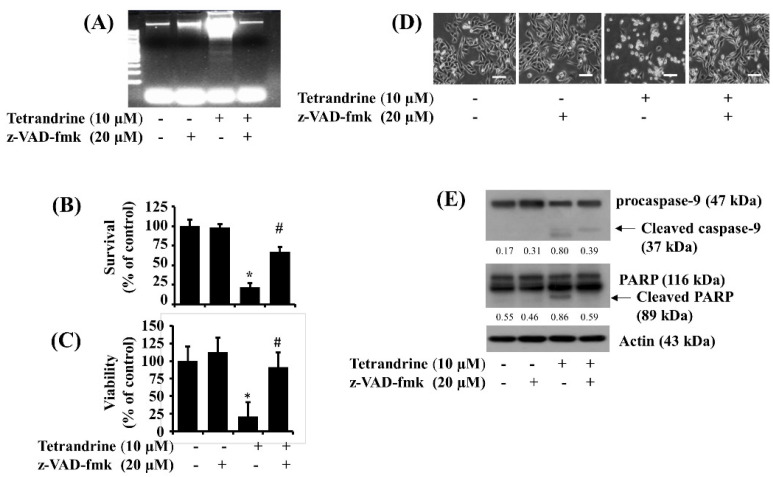
Effects of z-VAD-fmk on the tetrandrine-induced apoptosis, growth suppression, and altered expression of procaspase-9 and PARP in SW872 cells. (**A**–**E**) SW872 cells were pre-treated with 20 μM z-VAD-fmk for 1 h and then treated with vehicle control (0.1% DMSO) or tetrandrine at 10 μM for an additional 24 h. Genomic DNA from the conditioned cells was extracted and visualized by 1.8% agarose gel electrophoresis (**A**). The number of surviving cells was determined by cell count assay (**B**). Data are mean ± SE of three independent experiments. The cell survival was normalized as a percentage of control value without any drug. * *p* < 0.05 compared to the control value at the indicated concentration. # *p* < 0.05 compared to the value obtained from tetrandrine treatment in the absence of z-VAD-fmk. The viability of cells was analyzed by the MTS assay (**C**). The MTS assay was carried out in triplicate. Data are expressed as mean ± SE of three individual experiments. * *p* < 0.05 compared to the control value at the indicated concentration. # *p* < 0.05 compared to the value obtained from tetrandrine treatment in the absence of z-VAD-fmk. The morphological image of the conditioned cells was captured by a phase-contrast microscope (400×, scale bar = 50 μm). Each image is the representative of three independent experiments (**D**). Whole-cell lysates from the conditioned cells were prepared and analyzed by Western blotting (**E**) to measure the expression of procaspase-9, PARP, or β-actin. The image is the representation of three independent experiments.

Given that tetrandrine induces activation of caspases in SW872 cells herein, we next investigated the role of caspases in the tetrandrine-induced growth suppression and apoptosis of SW872 cells by using z-VAD-fmk (20 μM), a pan-caspase inhibitor. As shown in Figure 3A, z-VAD-fmk strongly blunted the tetrandrine-induced genomic DNA fragmentation in SW872 cells. In addition, z-VAD-fmk significantly inhibited the tetrandrine-induced reduction of SW872 cell survival and viability (Figure 3B,C), respectively. Microscopic observation further confirmed the ability of z-VAD-fmk to dramatically block the tetrandrine-induced reduction of SW872 cell survival (Figure 3D). Moreover, as shown in Figure 3E, z-VAD-fmk effectively suppressed the tetrandrine-induced generation of cleaved caspase-9 and PARP in SW872 cells. Expression levels of control actin protein remained unchanged under these experimental conditions. 

### 3.4. Tetrandrine at 10 μM Down-Regulates XIAP Expression at the Protein Levels in SW872 Cells

**Figure 4:**
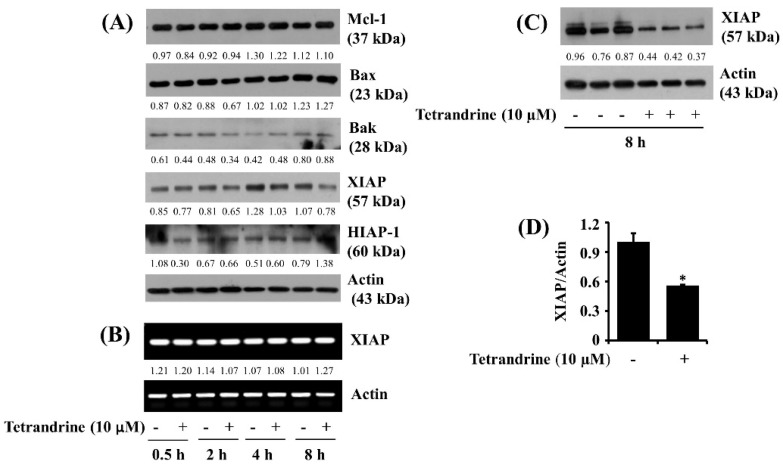
Effects of tetrandrine on the expression of the IAPs and Bcl-2 family members in SW872 cells. (**A**) SW872 cells were treated with vehicle control (0.1% DMSO) or tetrandrine at 10 μM for the indicated time point. Whole-cell lysates from the conditioned cells were prepared and analyzed by Western blotting to measure the expression of Mcl-1, Bax, Bak, XIAP, HIAP-1, or β-actin. (**B**) SW872 cells were treated with vehicle control (0.1% DMSO) or tetrandrine at 10 μM for the indicated time point. Total RNA from the conditioned cells was prepared and analyzed by RT-PCR analysis for measurement of the expression of XIAP or β-actin. (**C**) SW872 cells were treated with vehicle control (0.1% DMSO) or tetrandrine at 10 μM in triplicate for 8 h. Whole-cell lysates from the conditioned cells were prepared and analyzed by Western blotting for measurement of the expression of XIAP or β-actin. (**D**) Densitometry data of (**C**). * *p* < 0.05 was compared to the value of tetrandrine-free control at the indicated concentrations.

We next studied the effect of tetrandrine (10 μM) on the expression of the family of Bcl-2 and IAPs, anti-apoptotic proteins, in SW872 cells. As shown in Figure 4A, data of kinetic studies from the Western blotting analysis revealed that treatment with tetrandrine at times tested did not alter the expression of Bcl-2 family proteins, such as Mcl-1, Bak, and Bax, in SW872 cells. Of interest, tetrandrine treatment at 4 or 8 h led to the reduced expression of XIAP, but it did not affect HIAP expression in SW872 cells. Distinctly, RT-PCR experiments, as shown in Figure 4B, demonstrated that tetrandrine treatment at times tested did not influence XIAP transcripts in SW872 cells. Immunoblotting data from triplicate experiments further illustrated the ability of tetrandrine to vastly down-regulate XIAP protein expression in SW872 cells (Figure 4C). Densitometric data of Figure 4C for the protein expression levels of XIAP normalized to those of control actin is further shown in Figure 4D. Control actin protein and mRNA expression levels remained unchanged under these experimental conditions. 

### 3.5. Tetrandrine at 10 μM Reduces STAT-3 Phosphorylation and Expression in SW872 Cells, and Knockdown of STAT-3 Significantly Decreases the Cell Survival

**Figure 5:**
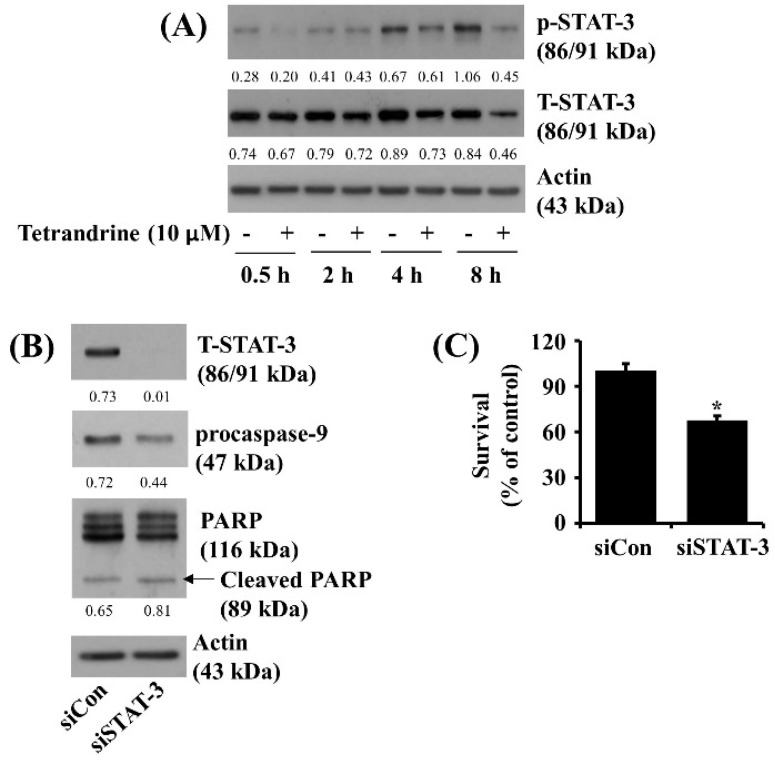
Effects of tetrandrine or knockdown of STAT-3 on the phosphorylation and expression levels of STAT-3, cell survival, procaspase-9, and PARP in SW872 cells. (**A**) SW872 cells were treated with vehicle control (0.1% DMSO) or tetrandrine at 10 μM for the designated time point. Whole-cell lysates from the conditioned cells were prepared and analyzed for measurement of the expression of p-STAT-3, STAT-3, or β-actin by Western blotting. p-STAT-3, phospho-STAT-3; T-STAT-3, total STAT-3. (**B**) SW872 cells were transfected with siRNA (siCon) and STAT-3 siRNA (siSTAT-3) for 24 h. Whole-cell lysates from the conditioned cells were prepared and analyzed to measure the expression of STAT-3, procaspase-9, PARP, or β-actin by Western blotting. (**C**) The number of surviving cells was measured by cell count assay. The cell count assay was carried out in triplicate. Data are expressed as mean ± SE of three individual experiments. * *p* < 0.05 was compared to the value of siCon at the designated time.

Evidence indicates that STAT-3 is highly phosphorylated and activated in many cancer cells [14], and its activation is closely linked to cancer cell survival [15]. This led us to investigate whether STAT-3 protein is expressed and phosphorylated in SAW872 cells and whether tetrandrine controls it. Notably, as shown in Figure 5A, kinetic studies revealed a time-dependent elevation of STAT-3 phosphorylation in SW872 cells. However, tetrandrine treatment at times tested led to intense repression of STAT-3 protein phosphorylation and expression in SW872 cells. Control actin protein expression levels remained constant under these experimental conditions. Using siRNA of STAT-3, we examined the role of STAT-3 down-regulation in the tetrandrine’s anti-survival effect on SW872 cells. As shown in Figure 5B, there was a complete loss of endogenous STAT-3 in STAT-3 siRNA-transfected SW872 cells compared with control siRNA-transfected cells. However, there was no or little effect on expression levels of procaspase-9 and cleaved PARP in the STAT-3 siRNA-transfected SW872 cells compared with control siRNA-transfected cells. As shown in Figure 5C, cell count analysis revealed that knockdown of STAT-3 caused a significant reduction in SW872 cell survival. 

### 3.6. Tetrandrine at 10 μM Up-Regulates Expression of GRP78 and ATF-4 and Phosphorylation of eIF-2α in SW872 Cells

**Figure 6:**
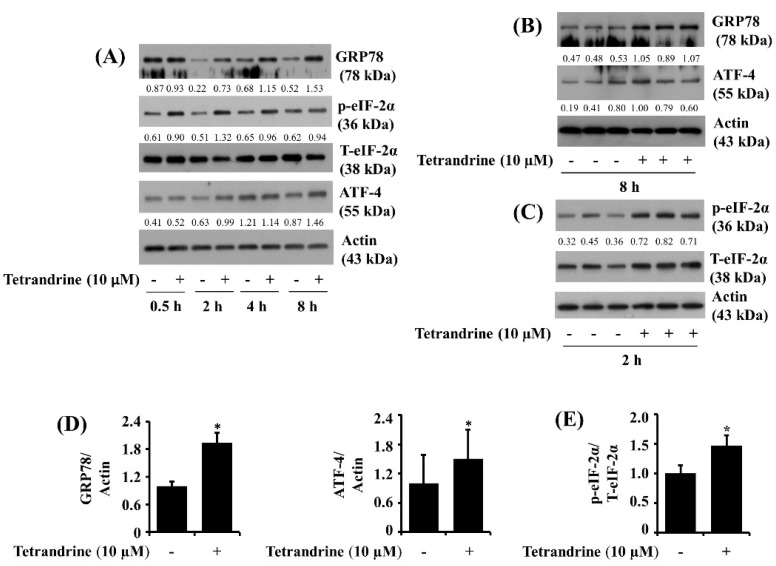
Effects of tetrandrine on the expression and phosphorylation of GRP78, eIF-2α, and ATF-4 in SW872 cells. (**A**) SW872 cells were treated with vehicle control (0.1% DMSO) or tetrandrine at 10 μM for the indicated time point. Whole-cell lysates from the conditioned cells were extracted and analyzed to measure the expression of GRP78, p-eIF-2α, eIF-2α, ATF-4, or β-actin by Western blotting. (**B**,**C**) SW872 cells were treated with vehicle control (0.1% DMSO) or tetrandrine at 10 μM tetrandrine in triplicate for 8 h (**B**) or 2 h (**C**). Whole cell lysates from the conditioned cells were extracted and analyzed to measure the expression of GRP78, ATF-4, p-eIF-2α, eIF-2α, or β-actin by Western blotting. (**D**,**E**) are densitometry data of (**B**,**C**), respectively. * *p* < 0.05 was compared to the value of tetrandrine-free control at the indicated concentrations.

We next analyzed the effect of tetrandrine on the expression and phosphorylation of ER stress and translation-related proteins, including GRP78, eIF-2α, and ATF-4, in SW872 cells. As shown in Figure 6A, compared with control, tetrandrine treatment at 2, 4, or 8 h resulted in high expression of GRP78 in SW872 cells. Moreover, tetrandrine treatment at 0.5, 2, or 4 h led to an elevation of eIF-2α phosphorylation with no change in total eIF-2α protein expression in SW872 cells. There was also ATF-4 protein up-regulation in SW872 cells treated with tetrandrine at 2 or 8 h. Control actin protein and mRNA expression levels remained unchanged under these experimental conditions. Western blotting data from triplicate experiments further confirmed the tetrandrine capability to significantly elevate the expression of GRP78 and ATF-4 (Figure 6B) and the phosphorylation of eIF-2α (Figure 6C) in SW872 cells. Densitometric data of Figure 6B,C for the expression of GRP78 and ATF-4 normalized to that of control actin and the phosphorylation of eIF-2α normalized to that of total eIF-2α is shown in Figure 6D,E, respectively. 

## 4. Discussion

Liposarcoma, a malignancy of adipocytes, is a rare but most common soft tissue sarcoma in adults [27]. It has been reported that poorly differentiated liposarcomas tend to commonly metastasize to the lungs and liver [28]. Tetrandrine is a bis-benzylisoquinoline alkaloid extracted from the roots of *Radix stephania tetrandrae* (S. Moore). It is known for the anti-cancerous effects of tetrandrine on many different human tumors [19,20,21,22,23,24,25,29,30]. Up to date, the tetrandrine regulation of growth of liposarcoma is not fully understood. Here we report that tetrandrine at 10 μM has strong anti-proliferative, anti-survival, and pro-apoptotic effects on SW872 human malignant undifferentiated liposarcoma cells by controlling the intrinsic caspase pathway, XIAP, STAT-3, and ER stress. 

In initial experiments, we have demonstrated the abilities of tetrandrine (10 μM) to significantly reduce the survival and viability of SW872 cells, pointing out its anti-proliferative and anti-survival effects. Considering the present findings that treatment with tetrandrine for 24 h also significantly inhibited the growth of 93T449 cells but was not cytotoxic to normal HaCaT human skin keratinocytes, it is conceivable that tetrandrine was not only effective to SW872 undifferentiated human liposarcoma cells but also effective to 93T449 differentiated human liposarcoma cells. These results may also imply that tetrandrine may exert its cytotoxicity to certain types of both undifferentiated and differentiated human liposarcoma cells.

Cells undergoing apoptosis have several biochemical characteristics, including genomic DNA fragmentation and the increase in the sub-G1 phase of cells [7]. Thus, assuming the present findings with the tetrandrine-induced nuclear DNA fragmentation in SW872 cells and the increased sub-G1 phase of the cells, it is evident that tetrandrine triggers apoptosis of SW872 cells and further exists its anti-cancer effect via induction of apoptosis. Apoptosis induction is mainly mediated through the extrinsic (death receptor) and intrinsic (mitochondrial) pathways [31]. In general, the extrinsic pathway is elicited through the up-regulation and activation of DRs on the surface of cells, which subsequently leads to the activation of downstream initiator caspase-8 [7]. On the other hand, the intrinsic pathway is triggered by alteration or damage of the mitochondrial integrity that is influenced by the expression of the Bcl-2 family proteins, which eventually leads to activation of downstream initiator caspase-9 [31]. Once activated, these caspase-8 and caspase-9 further activate the downstream effector caspases, such as caspase-3 and caspase-7. In resting cells, caspases are synthesized in inactive zymogen forms, but upon exposure to external apoptotic stimuli, they turn into active (cleaved) forms in cells undergoing apoptosis. The resultant active caspases participate in the execution of apoptosis by proteolytically cleaving their downstream substrates such as PARP and other vital proteins [9]. It is of interest demonstrated that activation of caspases is necessary for the tetrandrine-induced apoptosis in several tumor cells [20,21,32,33]. In this study, tetrandrine induces activation of the intrinsic, but not extrinsic, pathway in SW872 cells. Inactivation of caspases by z-VAD-fmk vastly abrogates the tetrandrine’s pro-apoptotic and anti-survival effects on the cells. These results suggest activating the intrinsic pathway is vital for the tetrandrine-induced apoptosis and growth suppression of SW872 cells. 

A notable finding of the present study is the tetrandrine regulation of XIAP and STAT-3 in SW872 cells. It has been reported that XIAP, a member of the IAPs family and an anti-apoptotic protein [34], has a critical role in apoptosis by directly inhibiting the activity of caspase-9 [35] and caspase-3 [36]. We have demonstrated that tetrandrine significantly down-regulates expression of XIAP, but not HIAP-1, another member of the IAPs family, at the protein level in SW872 cells. These results suggest that XIAP down-regulation by tetrandrine herein is due to a decrease in the protein stability and/or translational repression. The reduced XIAP expression may facilitate the tetrandrine-induced apoptosis and activation of caspase-9/3 in SW872 cells. Accordingly, the family of STATs, including STAT-3, is highly expressed and phosphorylated (activated) in a wide range of cancer cells and has an essential role in cell proliferation and survival [37]. Of further note, previous studies have demonstrated the dysfunctional activation of STAT-3 in cancer [38,39,40] and the significance of STAT-3 activation in abnormal cell proliferation and malignant transformation [41]. These findings strongly imply that inhibition and/or knockdown of STAT-3 could be a therapeutic target against cancers in which abnormal STAT-3 activation and expression are problematic. Of interest, it has been recently demonstrated that STAT-3 inhibition induces apoptosis of different types of fibrosarcoma cells [42]. Of further note, in a recent study, we have demonstrated that STAT-3, but not STAT-5 (another STAT-family members) is substantially expressed and phosphorylated in SW872 cells and its expression and phosphorylation play pivotal roles in the cell survival and apoptosis inhibition based on the facts that STAT-3 gene silencing leads to the cell growth inhibition and apoptosis induction [42]. In this study, tetrandrine treatment markedly reduces STAT-3 expression and phosphorylation in SW872 cells. To our best knowledge, the first reporting the ability of tetrandrine to inhibit STAT-3 in liposarcoma cells. Of further importance, considering the present findings that gene silencing of STAT-3 leads to a partial decrease in the expression of procaspase-9, a slight increase in cleaved PARP, and a reduction of SW872 cell survival, it is likely that STAT-3 is indeed a survival factor and an anti-apoptotic protein in SW872 cells. The reduced STAT-3 expression and phosphorylation further contribute to the tetrandrine’s growth-suppressive and apoptosis-inducing effects on SW872 cells. It will be important to challenge, in the future, whether overexpression of STAT-3 will block or interfere with the tetrandrine’s anti-survival and/or pro-apoptotic effects on SW872 cells to confirm the role of STAT-3 transcription factor in this process.

It is known that ER stress plays an essential role in inducing apoptosis of cancer cells in response to anti-cancer drugs or agents [13,43]. There are several characteristics in cells undergoing ER stress, including up-regulation of chaperon proteins such as GRP78, hyperphosphorylation (inhibition) of eIF-2α, and overexpression of ATF-4 [44,45,46]. Accordingly, tetrandrine is an inducer of ER stress [47] Little is known about tetrandrine regulation of ER stress in liposarcoma cells. Interestingly, we have further shown the ability of tetrandrine to elicit ER stress in SW872 cells, as evidenced by both up-regulation of GRP78 and ATF-4 and eIF-2α hyperphosphorylation. Assuming that eIF-2α hyperphosphorylation and ATF4 overexpression under ER stressful conditions leads to global translation inhibition, it is likely that the tetrandrine anti-survival and pro-apoptotic effects on SW872 cells are further attributable to triggering ER stress and inhibiting protein synthesis. 

**Figure 7:**
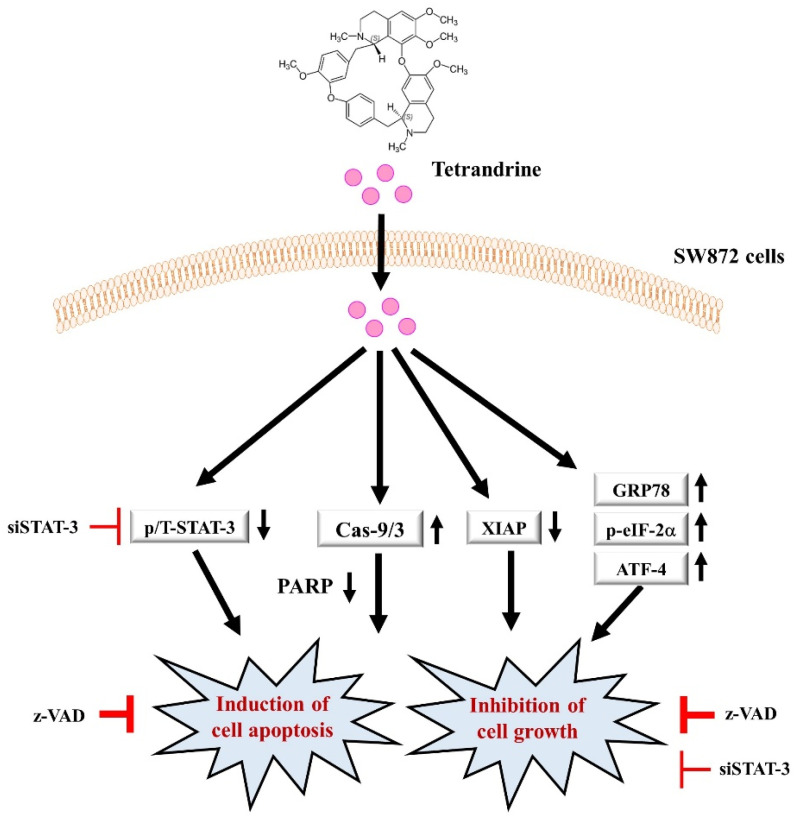
A diagram of the tetrandrine’s anti-survival and pro-apoptotic mechanisms (signaling pathways) in SW872 cells. Tetrandrine may activate the intrinsic (caspase-9/3-dependent) apoptotic pathway, which is crucial for the drug’s apoptosis-inducing and growth-suppressive effects on SW872 cells. In addition, tetrandrine may further inhibit STAT-3 and down-regulate XIAP, which may also confer the drug’s anti-survival and probably pro-apoptotic properties. Moreover, tetrandrine may induce the GRP78/ATF-4/eIF-2α-dependent ER stress and/or global translational inhibition, which could further contribute to the drug’s anti-survival and pro-apoptotic effects on SW872 cells.

It is proposed that a likely scenario of the molecular and cellular mechanisms underlying the tetrandrine’s anti-growth and pro-apoptotic effects on SW872 cells herein is that (1) tetrandrine induces activation of the intrinsic (caspase-9/3-dependent) apoptotic pathway, which is crucial for this natural substance’s apoptosis-inducing and growth-suppressive effects on SW872 cells, (2) tetrandrine lowers the expression levels of STAT-3 and XIAP in SW872 cells, which further contribute to the drug’s anti-survival and probably pro-apoptotic properties, and (3) tetrandrine elicits the GRP78/ATF-4/eIF-2α-dependent ER stress and/or global translational inhibition, which also confer the drug’s anti-survival and pro-apoptotic effects on SW872 cells (Figure 7).

In summary, we demonstrate for the first time that tetrandrine has strong growth-suppressive and apoptosis-inducing effects on SW872 human malignant liposarcoma cells. These effects are mediated through activation of the intrinsic caspase pathway, down-regulation of XIAP and STAT-3, and triggering ER stress. This work advocates that tetrandrine may be used as a potential anti-liposarcoma agent.

## Data Availability

Data is contained within the article. All data are available upon request from the corresponding author.

## Supplementary Materials

The following supporting information can be downloaded at: https://www.mdpi.com/article/10.3390/biom12060843/s1, Figure S1: Effects of tetrandrine on the survival and viability of 93T449 and HaCaT cells; Table S1: The list of antibodies used for Western blot analysis; Table S2: The primer sequences used for RT-PCR analysis.
