# Supplementary material for: Growth-Suppressive and Apoptosis-Inducing Effects of Tetrandrine in SW872 Human Malignant Liposarcoma Cells via Activation of Caspase-9, Down-Regulation of XIAP and STAT-3, and ER Stress
Source: Biomolecules. 2022 Jun 17;12(6):843. doi: 10.3390/biom12060843 (PMC9221093; doi:10.3390/biom12060843)

Table S1: List of antibodies used for Western blot analysis.

Antibodies	Dilution used	Source	Catalog no.
Primary antibodies			
Procaspase-3	1:2,000	Enzo Life Sciences	ADI-AAP-113
Procaspase-9	1:2,000	Enzo Life Sciences	ADI-AAP-139
PARP	1:2,000	Cell signaling	#9532
DR-5	1:2,000	IMGENEX	IMG-120E
Mcl-1	1:2,000	Santa Cruz Biotechnology	sc-819
Bax	1:2,000	Santa Cruz Biotechnology	sc-493
Bak	1:2,000	Santa Cruz Biotechnology	sc-1035
XIAP	1:2,000	B&D systems	AF8221
HIAP-1	1:2,000	B&D systems	AF8171
p-eIF-2α (S51)	1:2,000	Abcam	ab32157
T-eIF-2α	1:2,000	Cell signaling	#9722
GRP78	1:2,000	Santa Cruz Biotechnology	sc-13968
ATF-4	1:2,000	Santa Cruz Biotechnology	sc-200
p-STAT-3 (Y705)	1:2,000	Santa Cruz Biotechnology	sc-8059
T-STAT-3	1:2,000	Santa Cruz Biotechnology	sc-8019
Secondary antibodies			
Goat anti-rabbit IgG-HRP	1:2000	Santa Cruz Biotechnology	sc-2004
Goat anti-mouse-IgG-HRP	1:2000	Santa Cruz Biotechnology	sc-2005
Donkey anti-goat-IgG-HRP	1:2000	Santa Cruz Biotechnology	sc-2033

Table S2: Sequences of primers used for RT-PCR.

Gene	Forward	Reverse
XIAP	CGTCGATTTTTGTGATGCTCGTCAG	GAAGCATTTATCAGGGTTATTGTCTCATG
Actin	TCAAGATCATTGCTCCTCCTG	CTGCTTGCTGATCCACATCTG

Figure S1: Effects of tetrandrine on the survival and viability of 93T449 and HaCaT cells

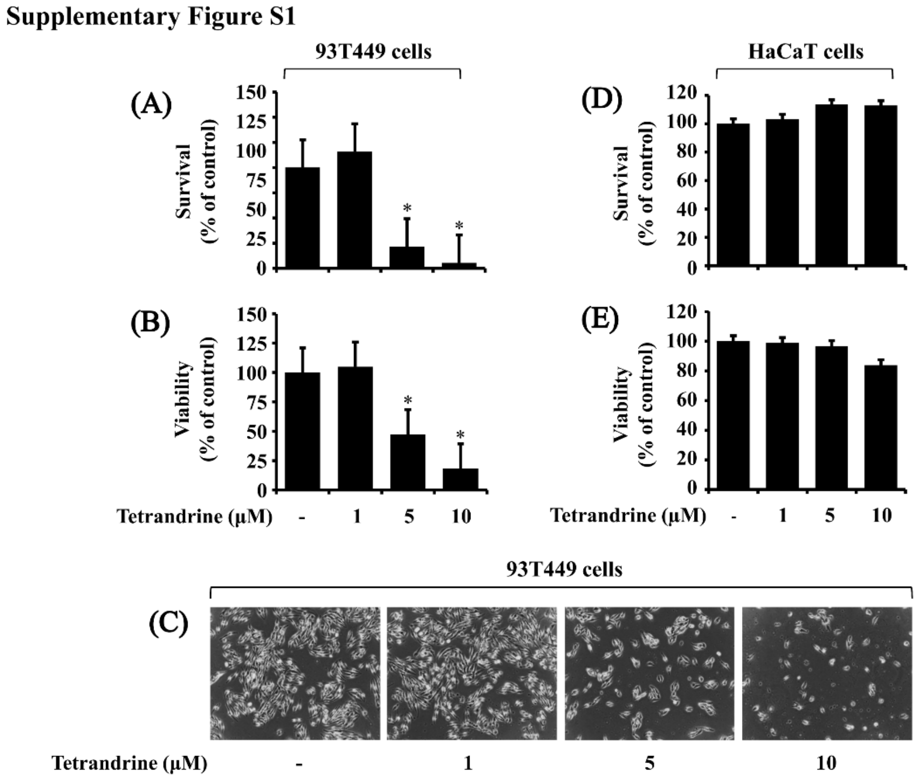

Supplement: Supplementary file 1 [file biomolecules-12-00843-s001.zip › biomolecules-1676489-supplementary.pdf]
